# Comparison of safety and efficacy of posterior lumbar interbody fusion (PLIF) and modified transforaminal lumbar interbody fusion (M-TLIF) in the treatment of single-segment lumbar degenerative diseases

**DOI:** 10.1186/s13018-024-04531-3

**Published:** 2024-01-30

**Authors:** Ming Chen, Jianchao Cui, Youtao Liu, Zhuoyan Cai, Cekai Yang, Hao Liu, Yuan Chen, Zhensong Yao

**Affiliations:** 1 Guangzhou University of Traditional Chinese Medicine, Guangdong, China; 2 Department of Orthopedic, The First Affiliated Hospital of Guangzhou University of Chinese Medicine, No. 16, Airport Road, Baiyun District, Guangzhou, 510000 China; 3 Department of Orthopedic, Baiyun Hospital of the First Affiliated Hospital of Guangzhou University of Chinese Medicine, Helong 7 Road, Baiyun District, Guangzhou, 510000 China; 4 Department of Orthopedic, Zhanjiang First Hospital of Traditional Chinese Medicine, Zhanjiang, China; 5 Jiang Yang Urban Construction Vocational School Abstract, Luzhou, China

**Keywords:** Modified transforaminal lumbar interbody fusion, Posterior lumbar interbody fusion, Lumbar degenerative diseases, Postoperative complications, Adjacent segmental degeneration

## Abstract

**Objective:**

To compare modified transforaminal lumbar interbody fusion (M-TLIF) with posterior lumbar interbody fusion (PLIF) in the treatment of single-segment lumbar degenerative disorders in order to assess its safety and effectiveness.

**Methods:**

From January 2016 to January 2021, 74 patients who received single-segment M-TLIF were examined. A total of 74 patients having single-segment PLIF during the same time period were included in a retrospective controlled study using the same inclusion and exclusion criteria. The two groups were compared in terms of the fusion rate, the Oswestry disability index (ODI), the visual analogue scale of low back pain (VAS), the perioperative condition, the postoperative complications, and the postoperative neighbouring segment degeneration.

**Results:**

All patients had surgery satisfactorily and were monitored for at least a year afterwards. The baseline values for the two groups did not significantly differ. The interbody fusion rate between PLIF (98.65%) and M-TLIF (97.30%) was not significantly different. In the follow-up, the M-TLIF group's VAS score for low back and leg pain was lower than that of the PLIF group. The ODI score of the M-TLIF group was lower than that of the PLIF group at 7 days and 3 months following surgery. Both groups' post-op VAS and ODI scores for low back and leg pain were much lower than those from before the procedure. In M-TLIF group, the operation time, drainage tube extraction time, postoperative bed rest time and hospital stay time were shorter, and the amount of intraoperative blood loss was less. Compared with those before operation, the height of intervertebral space and intervertebral foramen were significantly increased in both groups during postoperative follow-up (*P* < 0.05). The postoperative complications and adjacent segment degeneration of M-TLIF were significantly lower than those of PLIF.

**Conclusions:**

M-TLIF is a safe and effective treatment for lumbar degenerative disorders, with a high fusion rate and no significant difference between M-TLIF and PLIF. M-TLIF's efficacy and safety are comparable to that of PLIF, particularly in terms of early relief of low back pain and improvement in quality of life following surgery. Therefore, M-TLIF technology can be popularized and applied in clinic.

## Introduction

Lumbar degenerative disease is a kind of syndrome that can cause low back pain and other symptoms. It can also be characterized by progressive weakness, numbness, or intermittent claudication of the lower extremities. It seriously affects daily life and work, and can reduce the quality of life of patients and cause problems [1]. The common lumbar degenerative diseases are lumbar disc herniation, lumbar spinal stenosis, lumbar spondylolisthesis, scoliosis and so on [2]. At present, for lumbar degenerative diseases, patients with mild clinical symptoms are mainly treated with conservative treatment, but for lumbar degenerative diseases with severe pain, long-term conservative treatment is ineffective, seriously affect daily life, often need surgical treatment [3]. Lumbar fusion surgery is considered to be an effective treatment, which can significantly improve patients' symptoms such as low back and leg pain, and improve their quality of life [4].

Interbody fusion includes anterior approach, posterior approach, lateral approach, intervertebral foramen approach and so on. The most common posterior approach is posterior lumbar interbody fusion (PLIF) and transforaminal lumbar interbody fusion (TLIF) [5]. PLIF was first proposed by Briggs and Milligan [6] in 1944. After years of clinical application, it has been very mature in the treatment of degenerative lumbar diseases, such as lumbar spinal stenosis and lumbar spondylolisthesis [7]. PLIF technology is through the posterior median approach to separate muscles and other tissues from both sides of the spine, and then open the lamina, expose the spinal canal, decompress the nerve structure, and the implantation of pedicle screws becomes visual [8]. Because of the wide exposure range, wide field of vision and more thorough decompression of nerve root and dural sac, the success rate of operation is improved. But at the same time, PLIF has more damage to the surrounding tissue and more postoperative complications, which will also affect the stability of the lumbar spine after operation [9]. TLIF is a surgical method through intervertebral foramen approach to remove unilateral facet joints of vertebral lamina on the diseased side to realize spinal canal decompression and vertebral body fusion [10]. Because there are few spinal appendages to be removed, the stability of the spine is good, and the resection site is below the superior intervertebral nerve root during the operation, the nerve root exposure is easier, and can avoid excessive traction of dural sac and nerve root, complete full decompression of nerve root canal while reducing the risk of nerve root injury [11], it makes up for the deficiency of PLIF to some extent. However, unilateral resection of partial facet joints, especially unilateral facet joint bone graft fusion, may destroy the stability of internal fixation segments, especially in the case of torsional stress load [12]. Considering the potential shortcomings of simple PLIF or TLIF, we propose an modified transforaminal lumbar interbody fusion (M-TLIF) based on TLIF. In this article, we will fully evaluate the efficacy and safety of the M-TLIF procedure.

## Research materials and methods

### Participants

We retrospectively analysed 74 patients who underwent single-segment M-TLIF in the Department of Spinal Orthopaedics, the first affiliated Hospital of Guangzhou University of traditional Chinese Medicine from January 2016 to January 2021, and compared 74 patients with single-segment PLIF in the same period according to the same criteria. The following are the inclusion criteria: Lumbar disc herniation, lumbar spinal stenosis, and lumbar spondylolisthesis were diagnosed by orthopaedic surgeons as the source of low back pain or lower limb radiation numbness and pain. After 3 months of conservative treatment, there was no evident remission or progression of the disease. The first lumbar fusion was performed at the first affiliated Hospital of Guangzhou University of traditional Chinese Medicine. Preoperative and postoperative examinations were completed in our hospital, with complete imaging data (lumbar X-ray, CT, MRI). The patient is over 18 years old and has a completely independent ability of informed consent. The follow-up period was more than 1 year. Exclusion criteria are as follows: scoliosis; lumbar spondylolysis, spinal infectious diseases, spinal tumours or metastases, previous lumbar surgery, lumbar spondylolisthesis above grade I, ankylosing spondylitis, and patients with severe infection. Our research passed the hospital's ethical review.

### General information

We collected baseline data of eligible patients, including age, sex, body mass index (BMI), education, bone mineral density, course of disease, symptoms before the first operation, diagnosis before the first operation, vascular plaque before the first operation, smoking, alcohol consumption, hypertension, diabetes, chronic kidney disease, history of cerebrovascular disease, long-term hormone use, and walking distance before the first operation, whether the disease has progressed in the past 3 months.

### Operation procedure

Both groups were completed by the same team, improved preoperative examination, general anaesthesia under endotracheal intubation, routine disinfection and towel laying in the operation area. Taking L4-5 single-segment operation as an example, the following two surgical methods are introduced.

### M-TLIF

The prone position and the empty abdomen were taken, and the L4-5 intervertebral space was located under the fluoroscopy of Mobile C-Arm X-ray Equipment. The skin, subcutaneous and lumbar dorsal muscle fascia were cut open successively by the posterior median incision to expose the spinous process, vertebral lamina and articular process. L4 and 5 pedicle screws were placed and fixed. The isthmus was preserved and a portion of the degenerative side's lamina was removed. On the degenerative side, the medial 3/4 of the inferior articular process of L4 and the medial 1/4 of the superior articular process of L5 were removed; on the contralateral L4, the lateral 1/4 of the inferior articular process of L4 and the medial 1/4 of the superior articular process of L5 were resected. Expose the lateral spinal canal, remove the edge of the lamina and ligamentum flavum, expose the outside of the dural sac and protect the dura mater and nerve root during the operation. After the nerve root canal decompression, the nucleus pulposus was removed and the vertebral endplate cartilage was scraped. The autogenous cancellous bone and allogeneic bone were implanted into a suitable titanium alloy Cage interbody fusion cage to fix the intervertebral space. After re-confirming that there was no compression of nerve root and no obvious active bleeding. The articular surface of bilateral L4-5 facet joint was carefully repaired with nucleus pulposus forceps to create a bone graft bed, and autogenous cancellous bone and allogeneic bone particles were implanted into the bilateral articular surface to complete bilateral facet bone grafting. Once again, under the C-arm perspective, it is confirmed that the fixation is firm and there is no loosening. Rinse and place a drainage tube, suture the incision layer by layer and wrap it with aseptic excipients (Fig. 3).

### PLIF

According to the same method, the para spinal tissue was exposed and pedicle screws were implanted. The spinous process and lamina of L4 were removed completely with a bone knife, and the lamina was cut inward along the inner edge of the inferior articular process of L4. The width of the inferior articular process 3 ~ 5 mm and isthmus were preserved, and the cancellous bone was cut off. The ligamentum flavum at the lower edge of L4 lamina was removed with nerve stripping ions in order to remove the lamina and pay attention to protect the dural sac and avoid tear. Expose the bilateral nerve roots and treat the intervertebral disc to scrape off the endplate cartilage. After cartilage plate cleaning, the intervertebral space was exposed, autogenous bone particles and allogeneic bone were implanted into a suitable titanium alloy Cage interbody fusion cage, and the intervertebral space was fixed under pressure. Make sure the fixed position is good again and close the wound in the same way.

### Postoperative treatment

The postoperative treatment methods were the same in both groups. Prophylactic intravenous infusion of antibiotics for 1–2 days and intravenous drip of 5 ~ 10 mg/d dexamethasone for 3–5 days. Intravenous or oral analgesics were given according to the pain condition of the patients. According to the condition of the patients, absolute bed rest for 2–5 days after operation, ankle pump exercise and axis turning were performed under the guidance of doctors during bed rest to prevent the occurrence of bed rest complications. Get out of bed gradually under the guidance of an orthopaedic surgeon. Remove the drainage tube according to the drainage condition of the incision. When getting out of bed within 3 months after operation, it is necessary to strictly wear waistline to restrict waist activity, avoid sedentary and strenuous exercise, remove waistline and begin normal activity after 3 months.

### Evaluation

#### Related indexes during operation

Including operation segment, operation time, intraoperative blood loss, decompression intervertebral disc volume, postoperative extubation time, hospital stay and so on.

### Imaging index

**Fig. 1:**
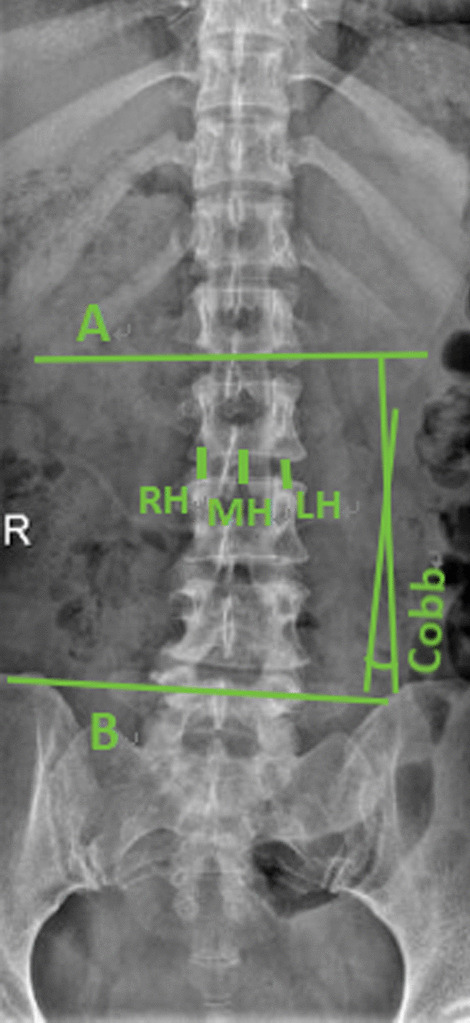
Measurement of coronal Cobb angle and height of intervertebral space on coronal X-ray of lumbar spine. The coronal Cobb angle (C-Cobb) refers to the angle between two straight lines perpendicular to A and B, A is the parallel line to the most inclined vertebral endplate at the head end, and B is the parallel line to the caudal most inclined vertebral endplate. The height of intervertebral space in coronal position (C-ISH) = (RH + MH + LH)/3, RH, MH and LH are the heights of right, middle and left intervertebral space, respectively

**Fig. 2:**
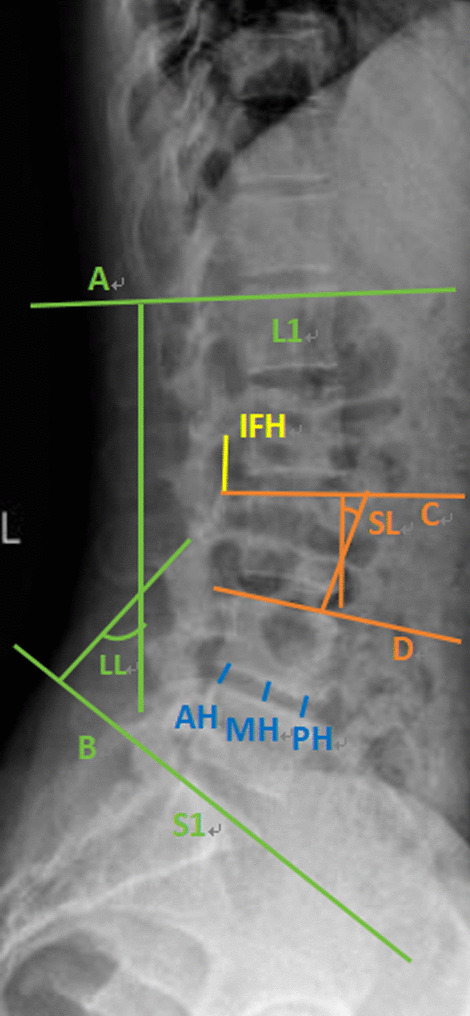
Measurement of relevant data on lateral X-ray. Lumbar lordosis angle (LL) [13] refers to the angle perpendicular to two straight lines A and B, A is parallel to the endplate of L1 vertebra, and B is parallel to the endplate of S1 vertebra. The segmental lordosis angle (SL) refers to the angle perpendicular to the two straight lines C and D, C is the parallel line to the upper endplate of the vertebral body, and D is the parallel line of the superior endplate of the inferior vertebral body. The intervertebral foramen height (IFH) is the vertical line between the lower edge of the pedicle of the upper vertebral body and the upper edge of the pedicle of the lower vertebral body, that is, a yellow straight line. The height of sagittal intervertebral space (S-ISH) = (AH + MH + PH)/3. RH, MH and LH are the heights of anterior, middle and posterior intervertebral space, respectively

**Fig. 3:**
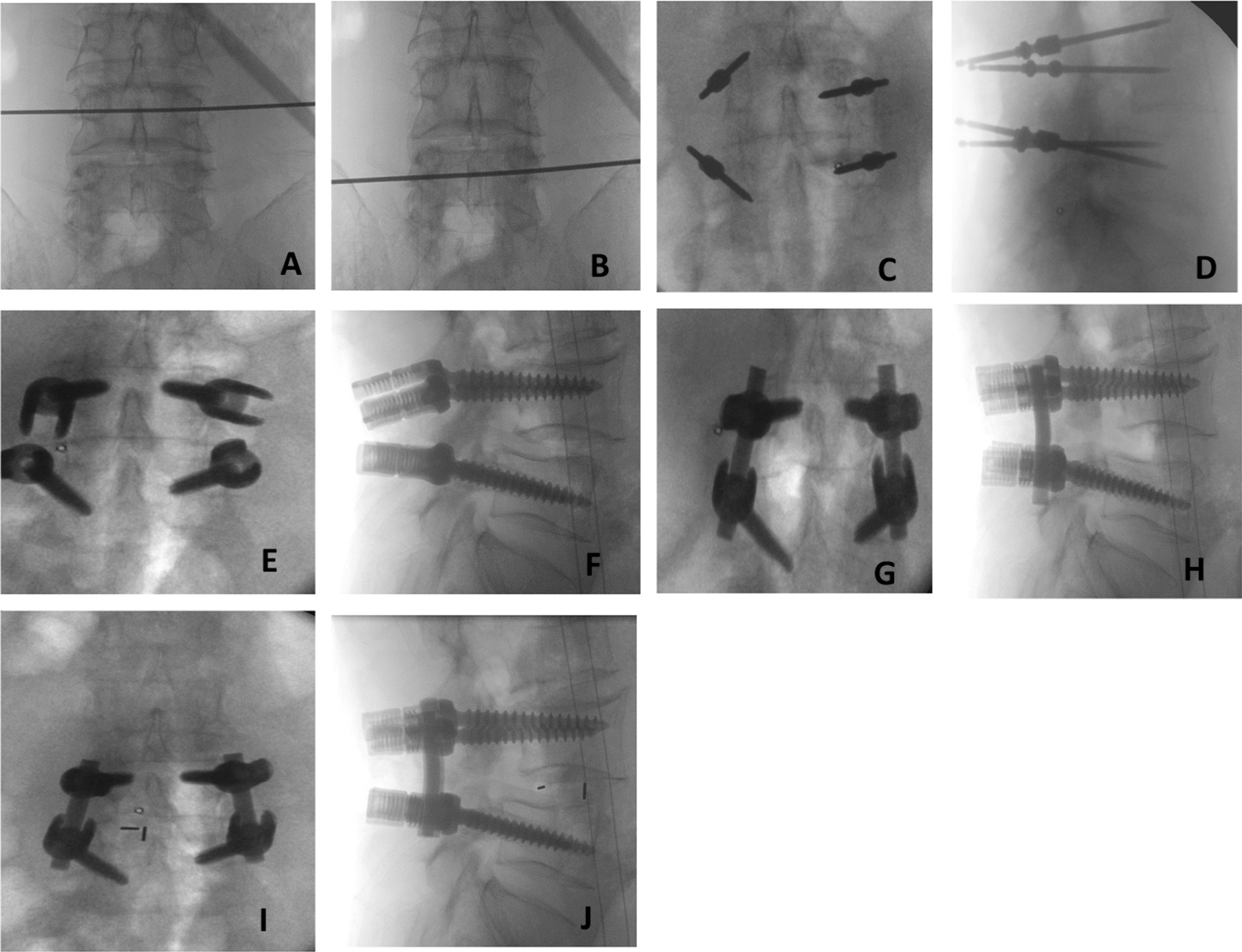
The procedure of M-TLIF operation. L4 (**A**) and L5 (**B**) vertebrae were identified under X-ray machine fluoroscopy; Kirschner wires were implanted along the pedicles of L4 and L5 vertebrae (**C**, **D**); After adjustment, pedicle screws of suitable size (**E**, **F**) were placed; suitable connecting rods were cut and fixed, laminae were opened, articular processes were cut off, and intervertebral discs were decompressed (**G**, **H**). A suitable interbody fusion cage (**I**, **J**) was filled from the degenerative side to restore the interbody height and the position of the cage was satisfactory

**Fig. 4:**
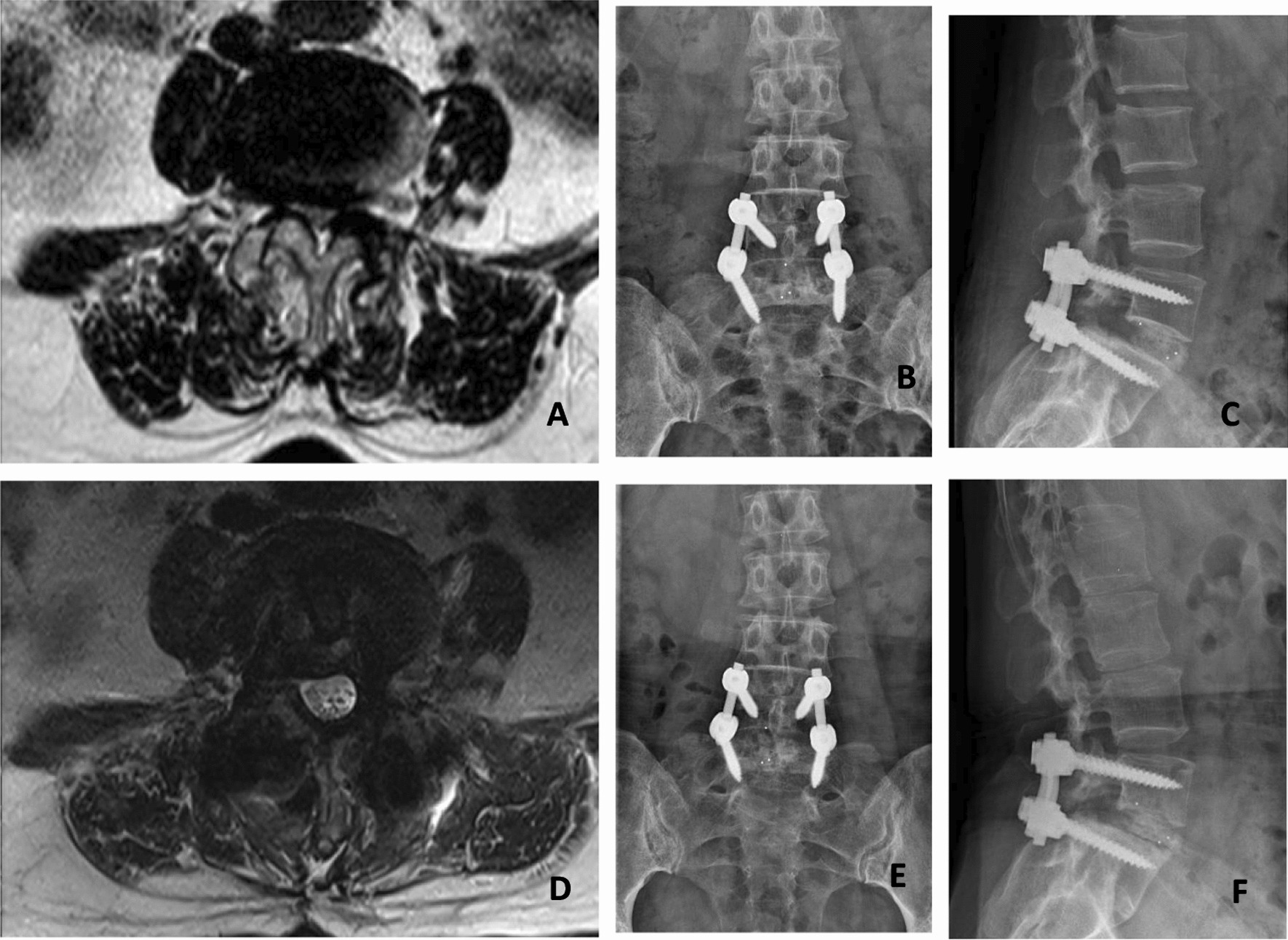
A 52-year-old woman complained of low back pain for more than 10 years, which was accompanied by right lower limb pain and numbness for 3 months. Before operation, axial MRI (**A**) showed lumbar disc herniation with lumbar spinal stenosis (L4/5). She underwent M-TLIF operation in our institution. The X-ray of anterior (**B**) and lateral (**C**) showed that the height of intervertebral space and intervertebral foramen returned to normal, and the position of interbody fusion cage was satisfactory. Axial MRI (**D**) showed that the spinal canal was significantly improved and the nerve root was decompressed completely. At the time of last follow-up, X-ray (**E**, **F**) shows that there is a clear and articular process fusion intervertebral fusion

The lumbar fusion rate and cage sinking rate were calculated at the last follow-up (Fig. 4). We used Bridwell grade to evaluate interbody fusion [14]. Grade I: bone trabeculae and upper and lower final plates are connected and the fusion cage is complete; II: bone trabeculae and upper and lower final plates are not fully connected, but the fusion cage is complete, and there is no gap between the fusion cage and the upper and lower final plates; III: the fusion cage is intact, and there is a gap between the fusion cage and the upper and lower final plate; IV: the fusion cage sinks, delayed fusion or fusion failure. The following indexes were measured before operation,6 months after operation and the last follow-up after operation (Figs. 1, 2, 3, 4): coronal Cobb angle (C-Cobb), lumbar lordosis angle (LL), segmental lordosis angle (SL), height of intervertebral space in coronal position (C-ISH), height of sagittal intervertebral space (S-ISH) and height of intervertebral foramen (IFH).

### Clinical effect evaluation

In this study, VAS score system [15] and ODI score system [16] were used to evaluate pain score and treatment effect before operation, 7 days after operation, 3 months after operation and at the last follow-up.

### Postoperative complication

It includes dural tear, postoperative stroke, postoperative infection, postoperative thrombus, screw loosening or fracture, intraspinal hematoma and so on.

### Degeneration of adjacent vertebral body

There are mainly lumbar disc herniation, lumbar spinal stenosis, lumbar spondylolisthesis and thoracolumbar fracture in adjacent segments after the first operation.

### Data analysis

We use SPSS25.0 software to analyse all the research data. All measurement data are judged by normality test first, the measurement data that accord with normality are expressed as average (standard deviation), while the measurement data that do not conform to normality are described by median (IQR), and the counting data are expressed by n (%). The measurement data with normality and uniform variance were compared by independent sample T test and paired sample T test. The data of skewness distribution were analysed by rank sum test (Wilcoxon test), and the counting data and grade data were analysed by chi-square test. When *P* < 0.05, there was significant difference between the two groups.

## Results

### Baseline clinical characteristics of the recruiters

We had a total of 148 participants, including 74 in the PLIF group and 74 in the M-TLIF group. The average age of all patients was 54.11 (13.02) years old (PLIF group 52.08 (13.50) years old, M-TLIF group 56.14 (12.28) years old). There was no significant difference in gender, age, BMI and other baseline data between the two groups (*P* > 0.05, Table 1).

**Table 1:** Comparison of clinical baselines

	PLIF (n = 74)	M-TLIF (n = 74)	T/Z/X^2^	*P*
Gender, n (%)			1.233	0.267
Male	17 (22.97%)	23 (31.08%)		
Female	57 (77.03%)	51 (68.92%)		
Age (years), mean (SD)	52.08 (13.50)	56.14 (12.28)	−1.911	0.058
BMI (kg/m^2^), mean (SD)	23.91(3.21)	24.18 (3.07)	−0.528	0.598
Degree, n (%)			1.565	0.211
Compulsory education	48 (64.86%)	55 (74.32%)		
University and above	26 (35.14%)	19 (25.68%)		
Bone mineral density (T value), mean (SD)	−1.40 (1.34)	−1.59 (1.32)	0.865	0.388
Course of the disease (years), median (IQR)	1 (1,4.25)	2 (0.58,6)	−0.494	0.621
Preoperative symptoms, n (%)			0.308	0.857
Low back pain	13 (17.57%)	12 (16.22%)		
Low back pain accompanied by pain in one lower limb	42 (56.76%)	40 (54.05%)		
Low back pain accompanied by pain in two lower extremities	19 (25.68%)	22 (29.73%)		
Follow-up time (months), median (IQR)	16 (12.75,18)	17 (13,21)	−1.896	0.058
Venous thrombosis, n (%)			0.695	0.706
No	71 (95.95%)	69 (93.24%)		
Neck	1 (1.35%)	1 (1.35%)		
Lower limb	2 (2.70%)	4 (5.41%)		
Smoking history, n (%)			0.499	0.480
No	62 (83.78%)	65 (87.84%)		
Yes	12 (16.22%)	9 (12.16%)		
History of drinking, n (%)			0.214	0.644
No	62 (83.78%)	64 (86.49%)		
Yes	12 (16.22%)	10 (13.51%)		
History of hypertension, n (%)			0.153	0.696
No	58 (78.38%)	56 (75.68%)		
Yes	16 (21.62%)	18 (24.32%)		
History of diabetes mellitus, n (%)			0.965	0.326
No	71 (95.95%)	67 (90.54%)		
Yes	3 (4.05%)	7 (9.46%)		
History of chronic kidney disease, n (%)				0.497*
No	74 (100%)	72 (97.30%)		
Yes	0	2 (2.70%)		
Chronic cerebrovascular disease, n (%)			0.174	0.677
No	70 (94.59%)	72 (97.30%)		
Yes	4 (5.41%)	2 (2.70%)		
Long-term hormone use (oral or intravenous), n (%)				0.120*
No	74 (100%)	70 (94.59%)		
Yes	0	4 (5.41%)		
Diagnosis, n (%)			0.87	0.647
Lumber disc herniation	9(12.16%)	13 (17.57%)		
Lumbar Spinal Stenosis	43(58.11%)	41 (55.41%)		
Lumbar spondylolisthesis	22(29.73%)	20 (27.03%)		
The state of disease in the past three months, n (%)			3.031	0.082
Almost	22(29.73%)	13 (17.57%)		
Aggravate	52(70.27%)	61 (82.43%)		
Walking distance before operation (m), n (%)				0.744*
> 1000 m	1 (1.35%)	0		
200–1000 m	19 (25.68%)	15 (20.27%)		
10–200 m	50 (67.57%)	54 (72.97%)		
≤ 10 m	4 (5.41%)	5 (6.76%)		

BMI, body mass index. *Results from fisher’s exact test. PLIF, Posterior lumbar interbody fusion. M-TLIF, Modified transforaminal lumbar interbody fusion. IQR, Interquartile range. SD, Standard deviation

### Comparison of perioperative data between PLIF group and M-TLIF group

Compared with PLIF group, M-TLIF group achieved shorter operation time, less intraoperative blood loss, postoperative drainage tube extraction time, postoperative bed rest time and hospital stay. For intraoperative disc decompression, PLIF showed a greater advantage (*P* < 0.05, Table 2). There was no significant difference in operation location, bone cement reinforcement, incision length and connecting rod length between the two groups (*P* > 0.05, Table 2).

**Table 2:** The perioperative condition of the two groups

	PLIF (n = 74)	M-TLIF (n = 74)	*P*
Operation location, n (%)			0.218*
L2/3	1 (1.35%)	0	
L3/4	1 (1.35%)	3 (4.05%)	
L4/5	43 (58.11%)	51 (68.92%)	
L5/S1	29 (39.19%)	20 (27.03%)	
Bone cement reinforcement, n (%)			0.492
No	68 (91.89%)	71 (95.95%)	
Yes	6 (8.11%)	3 (4.05%)	
Operation time (min), mean (SD)	180.51 (41.94)	159.34 (34.65)	0.001
Incision length (cm), mean (SD)	9.30 (1.98)	9.01 (1.85)	0.368
Intraoperative bleeding volume (ml), mean (SD)	186.49 (44.80)	132.16 (55.43)	0.001
Length of connecting rod (cm), mean (SD)	4.52 (0.76)	4.51 (0.42)	0.894
Decompressed intervertebral disc (ml), mean (SD)	7.30 (2.19)	6.14(1.76)	0.001
Extraction time of drainage tube after operation (days), mean (SD)	3.30 (1.40)	2.39 (0.77)	< 0.001
Postoperative bed rest time (days), mean (SD)	3.35 (0.63)	2.95 (0.66)	< 0.001
Length of stay (days), mean (SD)	12.19 (3.53)	10.87 (2.33)	0.008

*Results from fisher’s exact test. PLIF, posterior lumbar interbody fusion. M-TLIF, modified transforaminal lumbar interbody fusion

### Imaging results

In this study, it was observed that the interbody fusion rate was very high after M-TLIF (97.30%) and PLIF (98.65%), and there was no significant difference between the two groups (*P* > 0.05). Six months after operation and the last follow-up, the height of coronal and lateral intervertebral space in PLIF group was higher than that in M-TLIF group (*P* < 0.05, Table 3). Compared with those before operation, the height of coronal intervertebral space, lateral intervertebral space and intervertebral foramen were significantly increased in both groups 6 months after operation and at the last follow-up (*P* < 0.05, Table 3). In PLIF group, the coronal Cobb angle at 6 months after operation was significantly smaller than that before operation. There was no significant difference in other data (*P* > 0.05, Table 3).

**Table 3:** Imaging differences between the two groups

	PLIF (n = 74)	M-TLIF (n = 74)	*P*
C-Cobb (^o^), mean (SD)			
Preoperative	6.37 (4.71)	6.41 (4.69)	0.953
Six months after operation	5.50 (3.93)**	6.44 (5.05)	0.209
Last follow-up	5.76 (4.13)	6.23 (4.35)	0.503
LL (^o^), mean (SD)			
Preoperative	42.64 (12.83)	42.07 (12.16)	0.783
Six months after operation	43.16 (11.74)	42.95 (13.26)	0.920
Last follow-up	43.48 (12.66)	44.08 (13.39)	0.777
SL (^o^), Mean (SD)			
Preoperative	13.39 (6.79)	12.23 (6.86)	0.303
Six months after operation	13.67 (5.80)	12.27 (6.96)	0.185
Last follow-up	13.19 (6.89)	11.75 (6.39)	0.191
C-ISH (mm), mean (SD)			
Preoperative	9.33 (2.57)	8.95 (2.40)	0.361
Six months after operation	11.61 (1.86)**	10.87 (1.63)**	0.011
Last follow-up	11.63 (1.72)**	10.97 (1.71)**	0.022
S-ISH (mm), mean (SD)			
Preoperative	9.49 (3.16)	9.87 (2.41)	0.416
Six months after operation	12.50 (2.19)**	11.70 (1.78)	0.016
Last follow-up	11.87 (2.02)**	11.16 (1.64)**	0.020
IFH (mm), mean (SD)			
Preoperative	14.02 (4.32)	15.16 (4.10)	0.103
Six months after operation	16.93 (3.76)**	17.21 (3.68)**	0.650
Last follow-up	15.87 (3.65)**	15.99 (3.23)**	0.843
Fusion cage sinking, n (%)			0.071
No	62 (87.78%)	69 (93.24%)	
Yes	12(16.22%)	5 (6.76%)	
Interbody fusion, n (%)			1.000
No	1 (1.35%)	2 (2.70%)	
Yes	73 (98.65%)	72 (97.30%)	

**P < 0.05 compared to the preoperative data. PLIF, Posterior lumbar interbody fusion. M-TLIF, Modified transforaminal lumbar interbody fusion. C-Cobb, The coronal cobb angle; C-ISH, The height of intervertebral space in coronal position; LL, Lumbar lordosis angle; SL, The segmental lordosis angle; IFH, The intervertebral foramen height; S-ISH, The height of sagittal intervertebral space

### Clinical effect

At 7 days, 3 months and the last follow-up, the VAS score of low back pain and VAS score of leg pain in M-TLIF group were lower than those in PLIF group. At 7 days and 3 months after operation, the ODI score of M-TLIF group was lower than that of PLIF group. The VAS score for low back pain, leg pain, and ODI score of the two groups were significantly lower than those before the operation at 7 days after the procedure, 3 months after the operation, and the last follow-up after the operation (*P* < 0.05, Table 4).

**Table 4:** Comparison of clinical effects

	PLIF (n = 74)	M-TLIF (n = 74)	*P*
VAS of Low back, mean (SD)			
Preoperative	5.82 (1.01)	5.58 (0.92)	0.128
Seven days after operation	3.22 (0.88)**	2.80 (0.60)**	0.001
Three months after operation	2.35 (0.65)**	2.08 (0.36)**	0.002
Last follow-up	1.73 (0.50)**	1.43 (0.50)**	< 0.001
VAS of Leg, mean (SD)			
Preoperative	5.74 (0.88)	5.49 (0.74)	0.120
Seven days after operation	3.19 (0.96)**	2.69 (0.54)**	0.001
Three months after operation	2.38 (0.77)**	2.00 (0.43)**	0.002
Last follow-up	1.40 (0.53)**	1.31 (0.84)**	0.521
ODI, mean (SD)			
Preoperative	40.50 (3.12)	40.64 (3.10)	0.792
Seven days after operation	26.27 (4.11)**	23.95 (5.05)**	0.003
Three months after operation	17.85 (3.35)**	15.41 (4.40)**	< 0.001
Last follow-up	11.36 (2.40)**	11.08 (2.77)**	0.506

^**^P < 0.05 compared to the preoperative data. VAS, Visual analogue scale. ODI, Oswestry Disability Index. PLIF, Posterior lumbar interbody fusion. M-TLIF, Modified transforaminal lumbar interbody fusion

### Complications

In PLIF group, postoperative complications were 26 (35.14%) and 12 (16.22%), respectively. There was significant difference between the two groups. The incidence of intraspinal hematoma after PLIF was higher than that of M-TLIF (*P* < 0.05, Table 5), but there was no significant difference in other complications.

**Table 5:** Comparison of postoperative complications

	PLIF (n = 74)	M-TLIF (n = 74)	*P*
Dural tear, n (%)			1.000
No	72 (97.30%)	73 (98.65%)	
Yes	2 (2.70%)	1 (1.35%)	
Stroke, n (%)			1.000*
No	73 (98.65%)	74 (100%)	
Yes	1 (1.35%)	0	
Respiratory tract infection, n (%)			1.000*
No	74 (100%)	73 (98.65%)	
Yes	0	1 (1.35%)	
Urinary tract infection, n (%)			0.612
No	71 (95.95%)	73 (98.65%)	
Yes	3 (4.05%)	1 (1.35%)	
Deep venous thrombosis, n (%)			NA
No	74 (100%)	74 (100%)	
Yes	0	0	
The wound surface healed poorly n (%)			1.000
No	66 (89.19%)	67 (90.54%)	
Yes	8 (10.81%)	7 (9.46%)	
Deep infection at surgical site, n (%)			0.363
No	70 (94.59%)	73 (98.65%)	
Yes	4 (5.41%)	1 (1.35%)	
Screw loose or broken, n (%)			1.000
No	73 (98.65%)	73 (98.65%)	
Yes	1 (1.35%)	1 (1.35%)	
Intraspinal hematoma, n (%)			0.028*
No	68 (91.89%)	74 (100%)	
Yes	6 (8.11%)	0	
There are contralateral nerve root symptoms n (%)			1.000*
No	73 (98.65%)	74 (100%)	
Yes	1 (1.35%)	0	

*Results from fisher’s exact test. PLIF, Posterior lumbar interbody fusion. M-TLIF, Modified transforaminal lumbar interbody fusion

### Adjacent segment degeneration

The postoperative degeneration of adjacent segment in PLIF group was 45 (60.81%) and M-TLIF was 30 (40.54%). There was significant difference between the two groups. The possibility of osteophyte in the anterior edge of adjacent vertebrae after single-segment PLIF was higher than that in M-TLIF group (*P* < 0.05, Table 6).

**Table 6:** Comparison of adjacent segment degeneration

	PLIF (n = 74)	M-TLIF (n = 74)	*P*
Lumber disc herniation, n (%)			0.257
No	60 (81.08%)	65 (87.84%)	
Yes	14 (18.92%)	9 (12.16%)	
Lumbar Spinal Stenosis, n (%)			1.000
No	72 (97.30%)	73 (98.65%)	
Yes	2 (2.70%)	1 (1.35%)	
Spondylolisthesis, n (%)			0.612
No	73 (98.65%)	71 (95.95%)	
Yes	1 (1.35%)	3 (4.05%)	
Thoracolumbar fracture, n (%)			0.120*
No	74 (100%)	70 (94.59%)	
Yes	0	4 (5.41%)	
Osteophyte of anterior edge of vertebral body (> II grade), n (%)			0.029
No	62 (83.78%)	71 (95.95%)	
Yes	12 (16.22%)	3 (4.05%)	
Osteophyte of posterior margin of vertebral body, n (%)			0.366
No	66 (89.19%)	70 (94.59%)	
Yes	8 (10.81%)	4 (5.41%)	
Ligament calcification			1.000
No	69 (93.24%)	70 (94.59%)	
Yes	5 (6.76%)	4 (5.41%)	
Intervertebral disc cavity			1.000
No	72 (97.30%)	73 (98.65%)	
Yes	2 (2.70%)	1 (1.35%)	
Modic changes			1.000
No	73 (98.65%)	73 (98.65%)	
Yes	1 (1.35%)	1 (1.35%)	

*Results from fisher’s exact test. PLIF, Posterior lumbar interbody fusion. M-TLIF, Modified transforaminal lumbar interbody fusion

## Discussion

Lumbar vertebrae are the hub of human torso activities, which have many functions such as load-bearing, cushioning concussion, and exercise. Any activity will increase the burden of lumbar vertebrae [17, 18]. Therefore, long-term engaged in heavy physical labour, lumbar degeneration will become more obvious and serious, mainly lumbar disc herniation, lumbar spinal stenosis, lumbar spondylolisthesis and so on [19]. Typical symptoms are long-term lumbosacral pain accompanied by intermittent claudication, lower limb pain, weakness, progressive aggravation when walking, need to rest for a period of time to continue to walk, serious cases cannot walk at all, and even defecate dysfunction [20]. Studies have shown that surgical treatment is more successful than conservative treatment in patients with obvious lumbar symptoms [21, 22].

In recent years, the understanding of spinal structure and the in-depth exploration of biomechanics have greatly promoted the progress of spinal surgery. Lumbar interbody fusion includes anterior approach, posterior approach, lateral approach, transvertebral foramen approach and other methods. It is widely used in lumbar degenerative diseases such as lumbar spinal stenosis. Among them, PLIF and TLIF are commonly used surgical methods, especially in the treatment of lumbar disc herniation with lumbar spinal stenosis and so on [23, 24]. Both PLIF and TLIF can accurately expose the structure of the lesion, release the dural sac and nerve root, achieve spinal fusion by bone grafting on the basis of internal fixation, restore vertebral height and physiological kyphosis, and have a high fusion rate. PLIF has a wider field of vision and can better expose the nerve root. Posterior intervertebral bone grafting is performed while directly observing the nerve root and dural sac, which is very safe [25]. TLIF can complete interbody decompression and fusion under direct vision, the spine is stable, less accessory structure needs to be removed, less damage to lumbar structure, less bleeding during operation and less bed rest time after operation [26]. In this study, we describe a modified TLIF procedure, M-TLIF, for the treatment of single-segment lumbar degenerative diseases. In the course of operation, the extent of laminectomy and facet joint bone grafting are particularly important. In traditional TLIF surgery, the range of laminectomy is larger, and most of the diseased facet joints are removed, and facet joint bone grafting is often performed on the operative side. In our investigation, The isthmus was preserved and a portion of the degenerative side's lamina was removed. On the degenerative side, the medial 3/4 of the inferior articular process of L4 and the medial 1/4 of the superior articular process of L5 were removed; on the contralateral L4, the lateral 1/4 of the inferior articular process of L4 and the medial 1/4 of the superior articular process of L5 were resected, and performed bilateral facet joint bone grafting before the end of the operation.

In our study, both PLIF and M-TLIF achieved very good results. The long-term follow-up fusion rates of the two groups were very high, and there was no significant difference between the two groups, indicating that both PLIF and M-TLIF are safe and effective surgical methods for the treatment of single-segment lumbar degenerative diseases. Our results showed that in the early stage after operation, the VAS score of low back pain, VAS score of leg pain and ODI score of M-TLIF group were significantly better than those of PLIF group. At the last follow-up, there was no significant difference between the two groups. No matter what period after operation, the VAS score and ODI score of the two groups were significantly better than those before operation. This shows that both PLIF and M-TLIF can relieve the clinical symptoms of patients with single-segment lumbar degeneration, but M-TLIF has more advantages in the early stage after operation. This is closely related to the short time of M-TLIF operation, less bleeding during operation, less injury to spinal appendages during operation, short bed rest time after operation, and early low back muscle exercise in patients [27].

Martinelli et al. [28] retrospective analysis of 60 patients treated with PLIF or TLIF decompression; through measurement and calculation, there was no significant difference in Lumbar lordosis angle between the two surgical methods. In our study, similar results were obtained. There was no significant difference in postoperative Cobb angle, Lumbar lordosis angle and segmental lordosis angle between PLIF and M-TLIF groups. The restoration of intervertebral space height after PLIF is more advantageous than that of M-TLIF, which may be due to the maximum reduction and restoration of intervertebral space height during PLIF [29]. However, we also observed that in each group, the height of intervertebral space was larger than that before operation at 6 months after operation and at the last follow-up. This shows that both PLIF and M-TLIF can significantly restore the height of intervertebral space. PLIF and M-TLIF can have related complications, such as dural tear, postoperative stroke, surgical site infection, and contralateral nerve injury. The incidence of postoperative complications of PLIF and TLIF is not the same in different clinical studies [30]. In this study, the two groups of patients with postoperative dural tear, postoperative infection and other complications occurred, which can be recovered through related treatment. The incidence of complications after PLIF was higher than that of M-TLIF, especially intraspinal hematoma after PLIF. This may be due to a larger range of leakage during PLIF, and it is necessary to pull the dural sac to the opposite side when releasing the nerve root, which is easy to damage the nerve and increase the risk of cerebrospinal fluid leakage and intraspinal hematoma to some extent. Our study also observed that adjacent segment degeneration may occur after two kinds of surgery, including 45(60.81%) and 30(40.54%) cases in PLIF group and M-TLIF group at the last follow-up, respectively. This ratio is much larger than that of most previous studies [31–33]. A variety of reasons contribute to this result. The adjacent segment degeneration evaluated in our study is based on imaging, and we include more factors. In a recent study [34], we found that about 60% of patients had imaging degeneration of adjacent segments after single-segment lumbar fusion, which confirms our study to some extent. In our study, we found that osteophytes at the anterior edge of the vertebral body were more likely to occur in the adjacent segments after PLIF. We speculated that the position of the fusion cage was placed forward and the stress was more concentrated in the process of PLIF, which is worthy of further study.

Lumbar facet joint is an important part of spinal posterior column stability. Because of its narrow space and close contact between bone graft and surrounding bone, the fusion rate of lumbar facet joint is higher in theory. In our study, it is further confirmed that M-TLIF is not inferior to PLIF in clinical efficacy. In addition, M-TLIF has more advantages in early postoperative recovery, reduction of postoperative complications and postoperative adjacent segment degeneration. We estimate that M-TLIF will have a broad clinical application prospect for lumbar degenerative diseases in the future. If it can be widely used in clinic, it will reduce the tissue injury around the patients and improve the fusion rate. In terms of mechanical strength, bilateral facet joint fusion can effectively reduce the stress of the internal fixation system. It reduces the incidence of fatigue fracture of internal fixation, prevents mechanical failure, and ensures the long-term effect after operation. However, at present, there is no relevant research on whether there is a difference in long-term prognosis between M-TLIF and PLIF. Systematic biomechanical studies and long-term clinical effects and complications still need to be further studied.

In this study, there are also the following limitations. First, although we strictly follow the inclusion and exclusion criteria in the case selection process, there may still be a risk of selection bias; second, our follow-up period is short, and we expect follow-up studies to further extend the follow-up period to assess long-term outcomes. In addition, we only compared the difference between M-TLIF and PLIF in the treatment of single-segment lumbar degeneration, not with other surgical methods.

## Data Availability

The datasets used and/or analysed during the current study available from the corresponding author on reasonable request.
